# Maintenance of Physical Function in Adult and Older Adult Mice Using Aerobic Exercise

**DOI:** 10.1093/geroni/igab046.3500

**Published:** 2021-12-17

**Authors:** Ted Graber, Megan Pajski, Christopher Byrd, Nainika Nadigama, Alyssa Fennel, Emily Seguin, Anna Seguin

**Affiliations:** East Carolina University, Greenville, North Carolina, United States

## Abstract

As we age, physical and neuromuscular function declines gradually. Exercise is a therapy to improve neuromuscular ability. Pre-clinical models are needed to understand molecular mechanisms contributing to age-associated functional decline and how exercise affects that downward trajectory. Our goal was to compare the differences in effects of two validated mouse models of endurance exercise designed to mimic human training studies: high intensity interval training (HIIT) and voluntary wheel running (VWR). We hypothesized that both adult and older adults (10 and 26 months old at end, respectively: 10m and 26m) would respond to both exercise regimens by improving or maintaining exercise/physical capacity, but that adult mice would benefit more. We randomly assigned male C57BL/6 mice into experimental groups: 10m: (VWR, HIIT, sedentary control, CON, n=8 per group), and 26m (VWR, n=8, HIIT, n=10). We measured functional ability (pre- and post-intervention) using CFAB (comprehensive functional assessment battery), our composite scoring system (grip strength, inverted cling, treadmill endurance, activity rate, rotarod), tracked body composition (EchoMRI), and measured muscle wet mass. We found that significant retention of ability (CFAB difference, repeated measures ANOVA, p<0.05) and fat percentage (ANOVA., %change: 10m: CON +125%, HIIT +101%, VWR +52%; 26m: VWR -42%, HIIT +26%, p<0.05) was promoted by both exercise modalities compared to control, and furthermore HIIT may have better efficacy in the adult versus the older mice. In conclusion, both exercises are valid models with derived benefits as expected in similar human studies. We anticipate future work using these models to undertake mechanistic investigations.

